# ProtView: A Versatile Tool for *In Silico* Protease Evaluation and Selection in a Proteomic and Proteogenomic Context

**DOI:** 10.1021/acs.jproteome.3c00135

**Published:** 2023-05-29

**Authors:** Sophia S. Puliasis, Dominika Lewandowska, Piers A. Hemsley, Runxuan Zhang

**Affiliations:** † Division of Plant Sciences, School of Life Sciences, University of Dundee, Dow Street, Dundee DD1 5EH, Scotland, UK; ‡ Information and Computational Sciences, The James Hutton Institute, Invergowrie, Dundee DD2 5DA, Scotland, UK; § Cell and Molecular Sciences, The James Hutton Institute, Invergowrie, Dundee DD2 5DA, Scotland, UK

**Keywords:** protease, enzyme, digest, in silico digestion

## Abstract

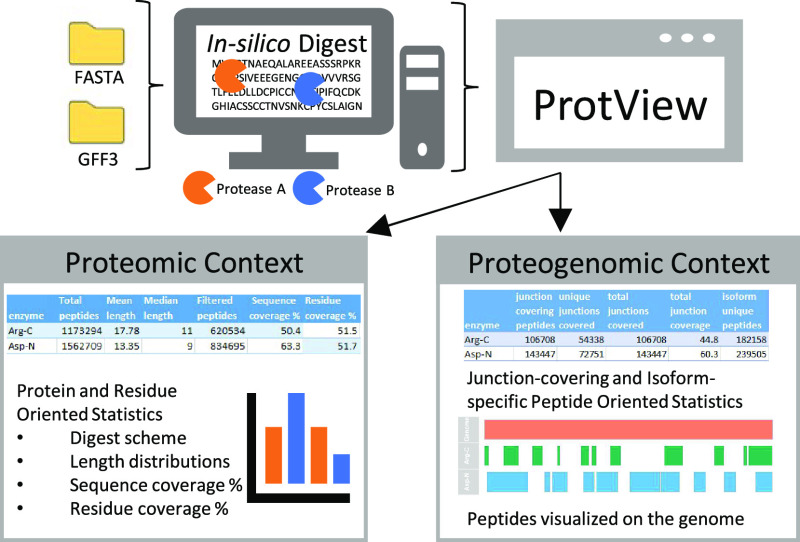

Many tools have been created to generate *in silico* proteome digests with different protease enzymes and provide useful information for selecting optimal digest schemes for specific needs. This can save on time and resources and generate insights on the observable proteome. However, there remains a need for a tool that evaluates digest schemes beyond protein and amino acid coverages in the proteomic domain. Here, we present ProtView, a versatile *in silico* protease combination digest evaluation workflow that maps *in silico*-digested peptides to both protein and genome references, so that the potential observable portions of the proteome, transcriptome, and genome can be identified. The proteomic identification and quantification of evidence for transcriptional, co-transcriptional, post-transcriptional, translational, and post-translational regulation can all be examined *in silico* with ProtView prior to an experiment. Benchmarking against biological data comparing multiple proteases shows that ProtView can correctly estimate performances among the digest schemes. ProtView provides this information in a way that is easy to interpret, allowing for digest schemes to be evaluated before carrying out an experiment, in context that can optimize both proteomic and proteogenomic experiments. ProtView is available at https://github.com/SSPuliasis/ProtView.

## Introduction

Bottom-up proteomics involves using proteases to digest protein mixtures into peptides, which are then analyzed by mass spectrometry (MS), allowing the peptides, and thus the originating protein, to be identified.^1^ Shotgun proteomics refers to the use of bottom-up methods to identify proteins in complex mixtures. The shotgun proteomics workflow typically begins with the protein sample being denatured, reduced, alkylated, and digested by one or more proteases into peptides, which are then separated by liquid chromatography and identified by tandem mass spectrometry (MS/MS) and database searching.^2^ Database searching determines whether a peptide sequence in a database gives a significant match to each MS/MS spectrum and the degree of matching is assigned a score.^3^ It is paramount that every step is carried out effectively to maximize individual peptide identification and quantification as this expands coverage and quantification accuracy at the protein level. When proteins are digested in the first stage, it is the resulting peptides that are carried into the subsequent analysis. Therefore, a peptide not generated in the digest or falling outside of the technical capabilities of the mass spectrometry setup cannot be identified in the subsequent analysis.

Trypsin is usually the protease of choice because it is highly specific, cleaving C-terminal to lysine (Lys) and arginine (Arg) residues,^4^ stable under a wide range of experimental conditions, and generates peptides in the preferred low charge and 7–35 amino acid length range for detection by MS machinery.^5^ However, it is not always the most suitable choice. For example, Lys and Arg are less frequent in membrane spanning protein regions,^6^ resulting in fewer detectable peptides per unit length, thus reducing identification and quantification using MS methods when integral membrane proteins are digested with trypsin. Lys and Arg also account for 25% of exon-ending and junction residues due to their codons (specifically Lys: AAG|^∨^, Arg: AG|G^∨^, AG|A^∨^. | denotes splice junction, ^∨^ denotes trypsin cleavage site) being encoded by the exon boundary of the splice site,^7^ resulting in trypsin cleavage at or across splice junctions. This impedes the detection of junction-spanning peptides necessary to identify splice isoforms arising from alternative mRNA splicing. Furthermore, due to enzyme specificity, the perpetual use of any highly specific protease will continuously generate the same subsets of peptides. This eventually leads to a ‘tunnel vision’ display of the proteome in databases and repositories, with regions or even whole proteins that do not produce MS/MS-suitable peptides with the protease in question remaining unidentified and uncharacterized.^8^

Digestion with different proteases, either to replace or to complement trypsin, has emerged as a way of mitigating the above issues and has proven to be useful in the study of membrane proteins,^9^ splice junctions,^7^ N-termini not accessible by trypsin,^10^ and post-translational modifications (PTMs).^11^ These explorations into the use of alternative proteases support the argument that there could be an ideal, non-trypsin centric, digestion scheme for every biological question and type of analysis.^8^

Expanding upon this idea, multiple protease digestion strategies can also be brought to bear on the issue of uncharacterized proteins. Combining multiple enzymes can be done in parallel, where peptide information from multiple different single protease digests (e.g., separate digests of the sample with trypsin and chymotrypsin) is combined during post MS/MS analysis, or concurrently, where multiple proteases are added to the same sample *in vitro* before MS/MS analysis is carried out. It has been reported that using enzymes in parallel results in a significant increase in sequence coverage compared to single digests of the *Saccharomyces cerevisiae* proteome,^5^*Cannabis sativa* buds,^12,13^ human cervical cancer cells,^14^ and human recombinant proteins.^15^ On the other hand, concurrent digests were reported to increase the number of identified proteins when trypsin-Asp-N was used on *Schizosaccharomyces pombe* whole cell lysates when compared to trypsin alone.^16^ Concurrent use of trypsin-Lys-C was also more efficient at yielding fully cleaved peptides and reducing the frequency of missed cleavage, compared to either protease alone on *S. cerevisiae* samples.^17^ In data-independent acquisition (DIA) approaches, multiple proteases can also be multiplexed by carrying out the digests separately and then pooling the peptides prior to MS/MS analysis. This multiplex approach has been shown to increase protein sequence coverage in multiple cell lines (HEK 293T, HSC6, SCC25)^18^ and phosphosite identification in activated brown adipocyte, although there is a risk of lowering peptide identifications due to increased sample complexity.^19^

The aforementioned studies can make digest scheme recommendations for specific species and types of experiments only after empirical analysis of each digest scheme *in vitro;* there is no *a priori* method for informing protease selection to suit a biological question. As the scope of proteomic analyses is very broad, knowing which digest scheme is better suited to an analysis beforehand can therefore save on costs, time, and resources. Programs such as PeptideCutter^20^ and Rapid Peptides Generator (RPG)^21^ can digest protein sequences with different enzymes *in silico* to give peptides that will theoretically be generated by a digest. ProteaseGuru^22^ and Proteogest^23^ go a step further and provide interpretations of their digest results to aid in protease selection. Proteogest is a Perl application that allows the user to select a combination of provided or custom modifications and to assess the effects of PTMs on the outcome. ProteaseGuru is a versatile and accessible tool that provides detailed peptide information and includes database PTM annotations and data visualization in the outputs. Nonetheless, there remains a need for a tool that can also provide information in a wider context that includes transcriptomic and genomic coverages and regions, to aid the study of alternative splicing and identify peptides that are unique to individual transcript isoforms, thus allowing the identification and quantification of the effects of transcriptional and translational regulations. Given the number of diseases caused or promoted by changes in splicing of proteins,^24^ the ability to detect splice isoforms could be used to understand disease mechanisms or in diagnostic settings.

This work introduces ProtView, a method that provides the set of possible peptides that can be identified by each protease, or protease combination, and the variable information that they present, such as peptide length distributions, protein sequence coverage, and amino acid coverage using *in silico* digestion. It also maps the digested peptides to the genome using coding sequence (CDS) information from the annotations, which can provide detailed locations and information of the digested proteome in transcriptomic and genomic context, enabling analyses such as the identification of splice junction covering peptides, and isoform-unique peptides. It is the first tool that allows for the use of *in silico* proteomic predictions to direct proteomic investigation of alternative splicing, polyadenylations, alternative transcriptional starting locations, and also alternative translational site regulations. We have demonstrated the utility of ProtView, with an analysis on the *Arabidopsis thaliana* proteome, revealing the most suitable digest schemes for achieving the highest coverage of protein sequence, splice junctions, and certain residues. We also show that ProtView can correctly predict the relevant performances of digestion schemes in most cases by comparing its results with *in vitro* protease experiments and published proteomic data. Through our analysis, we also identified that existing *in silico* digestion rules are not always accurate, which needs regular checking and revision based on experimental data to improve the accuracy of *in silico* coverage predictions.

## Methods

**Figure 1:**
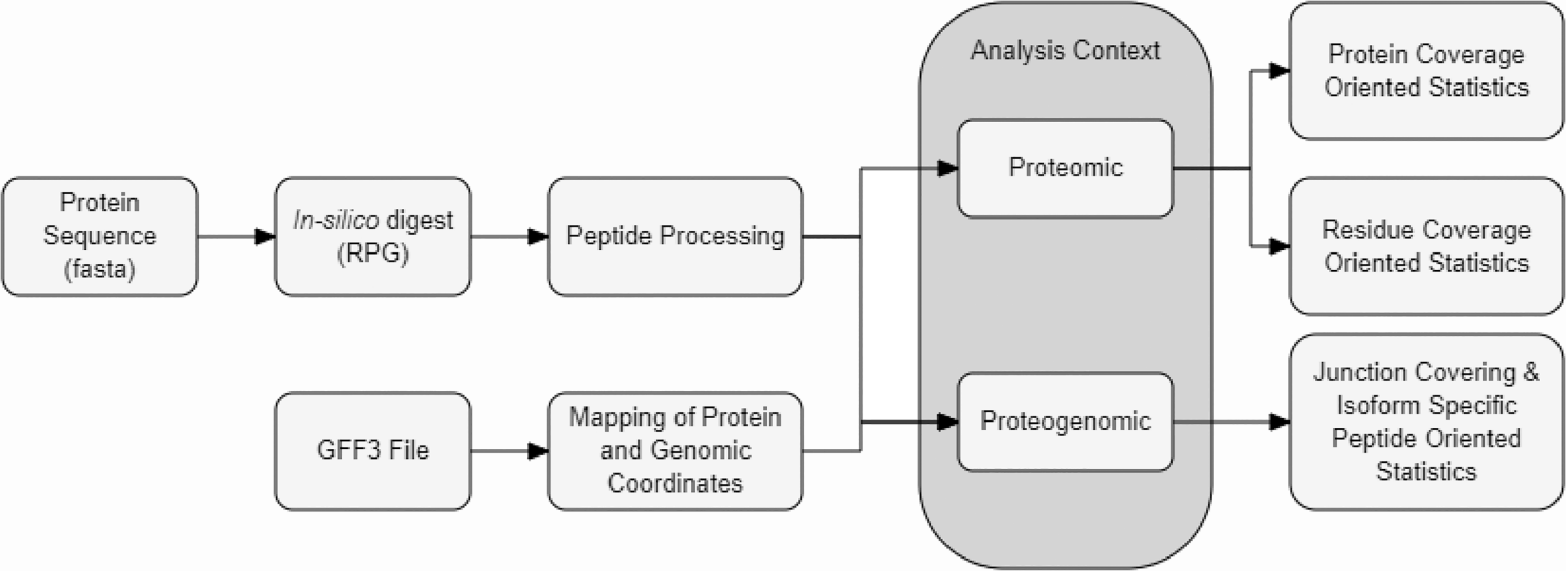
Outline of the ProtView workflow.

ProtView presents all the *in silico*-digested peptides from each protease, or protease combination, and detailed information on the proteome, transcriptome, and genome, such as peptide length distributions, protein sequence coverage, specific residue coverage, peptides that cover splice junctions, junction coverage, the number of isoform-unique peptides, and genomic coordinates of peptides (Figure 1). All programming was done in Python 3.8 under the GPL v3 license. Details of the program and instructions with tutorials can be found at https://github.com/SSPuliasis/ProtView.

### *In Silico* Digest

ProtView utilizes in silico digests carried out by Rapid Peptides Generator (RPG),^21^ which is incorporated into the ProtView installation. It can process the whole protein database in one go, allows for user-defined protease digestion rules, and can generate more information than PeptideCutter,^20^ such as isoelectric point of each peptide. RPG has the option to carry out single or concurrent digests, where a sequence is simultaneously cleaved by multiple enzymes. However, through investigation of the published *in vitro* data used in this study, our analysis shows that the digestion rules do not always accurately reflect what happens in the experiment; thus, we have revised the cleavage rules for Asp-N and Glu-C. The original Asp-N digestion rule in RPG cleaves N-terminal to both Asp and Cys, while our revised version only cleaves the N-terminal to Asp. Similarly, the original Glu-C digestion rule included in RPG cleaves the C-terminal to Asp and Glu, while our revised rule only cleaves the C-terminal to Glu. We have used revised versions (Asp-N-UD, Glu-C-UD) in all following analyses for Asp-N and Glu-C, and further data and discussion supporting the revision of digestion rules are provided in the results section. The revised protease digestion schemes are included with ProtView installation (Asp-N-UD, Glu-C-UD) allowing revised *in silico* digest to be carried out with RPG. Cleavage rules of all proteases used in *in silico* analyses can be found in Table S1.

### Peptide Processing

Using the digested peptides from RPG, ProtView can create parallel enzyme digests by combining peptides from individual single digests. Duplicates of peptides that are generated by different digests (e.g., by proteases with similar cleavage specificities) are removed from the parallel digest, resulting in a non-redundant parallel digest output. The enzyme names in the parallel digest output are the enzymes used separated by ’/’, whereas the enzyme names for concurrent digests are the enzymes used separated by ’-’. Peptides can then be filtered by amino acid length and/or by content of a specific residue or motif. Filtering for a length of 7–35 amino acids is recommended as the detectable range, although this optional filtering can be set by the user to match the instrumentation or criteria for the experiment. An example of a peptide generated by RPG and processed by ProtView can be found in Table S2.

### Proteomic Summary Statistics

Protein sequence coverage is the percentage of the original protein sequences that are covered by filtered peptides and is calculated as the ratio between the sum of amino acids covered by peptides and the total lengths of protein sequences in the FASTA file. Residue (or motif) coverage is the percentage of each amino acid (e.g., Thr in Table 1) in the original FASTA sequence file that is covered by the filtered peptides. The total numbers of peptides generated by each enzyme both before and remaining after filtering are also presented in a table format alongside their mean and median lengths. Table 1 is a summary table generated by ProtView for a single gene as an example, expandable to multiple genes or a proteome.

**Table 1:** Summary Statistics of a Digested Protein (AT3G48187) after Processing Digest Results, with Threonine (Thr) Coverage

enzyme	total peptides	mean length (aa)	median length (aa)	filtered peptides	sequence coverage (%)	Thr coverage (%)
Asp-N	39	21.7	17	25	55.9	51.0
Glu-C	56	15.1	9	29	52.8	36.7

### Genomic Coordinates of the Peptides

Using the genomic coordinates of coding sequences (CDSs) for each protein from gene and transcript annotations, ProtView assigns a unique ID to each CDS and the adjacent intron preceding it, calculates the length of each intron between CDSs, and converts the genomic coordinates of CDS regions to the relative protein sequence coordinates by taking the translation start and cumulative intron length into account. This information can then be used in both the conversion of peptide relative proteomic coordinates to genomic and in the identification of splice junction-covering peptides. An example of an extracted CDS can be found in Supplementary Table S3, and equations to convert the protein coordinates and genomic coordinates are shown in the Supporting Information.

**Figure 2:**
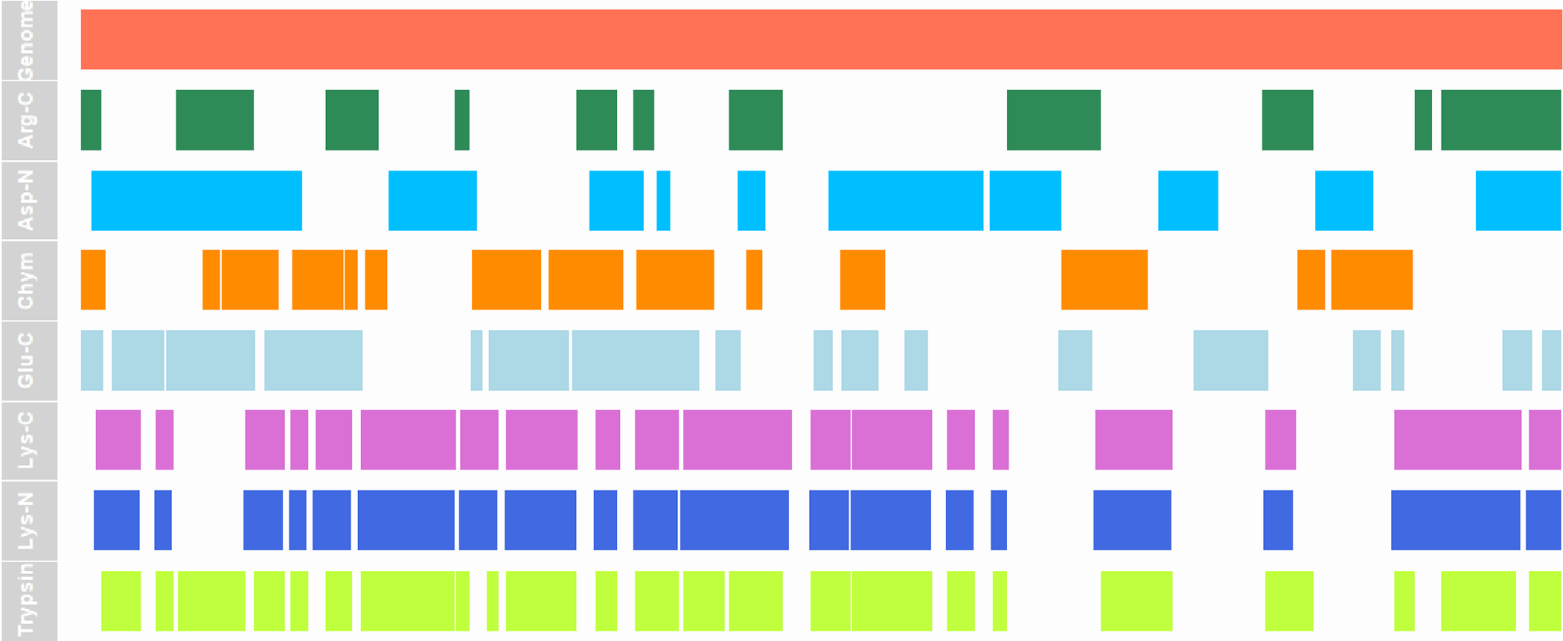
Gviz visualization of genome coverage for the 846 amino acid monoexonic gene AT3G48187.1 using peptides in the 7–35 aa range. The top row represents the genomic sequence. Regions of peptide sequence coverage generated by different proteases are shown in the subsequent rows.

The conversion of peptide coordinates to genomic is shown in Figure S1. The resulting data frame contains the parent isoform, both genomic and protein coordinates for each peptide, and the enzymes used to generate the peptide. Table 2 shows an example of two peptides (VDNVSARDDLVNVPPSDLISKLKLLAVGYNCSE, DSKFIGSPSICAGNSRKRFRKEWFRKFVSEV), generated by digestion of AT3G48187.1 with Glu-C and Asp-N, respectively, and their genomic coordinates. Note that the peptides share the relative protein coordinate 322 (Asp322) but that their genomic coordinates differ after the conversion due to ProtView giving the outer bound of genomic coordinates for the shared codon, depending on whether a coordinate is at the start or the end of a peptide (i.e., first or last base of the codon, in this case GAT). Genome browser and visualization tools such as the R package Gviz^25^ can be used to visualize peptides mapped onto the genome, as shown in Figure 2.

**Table 2:** Output Format of Genomic Coordinate Conversion Function of Two Exemplar Digested Peptides

peptide	isoform	protein start coordinate	genomic start co-ordinate	protein end coordinate	genomic end coordinate	enzyme
DNTYGSEMINFDSKFIGSPSICAGNSRKRFRKEWFRKFVSEV	AT3G48187.1	292	17,798,501	322	17,798,593	Asp-N
VDNVSARDDLVNVPPSDLISKLKLLAVGYNCSE	AT3G48187.1	322	17,798,591	354	17,798,689	Glu-C

### Junction Covering and Isoform Unique Peptide Identification and Statistics

Another function of ProtView is to identify peptides that span splice junctions. Using the conversion between genomic and proteomic coordinates we developed above, we can calculate the protein coordinates for each splice junction using the adjacent coding exon protein coordinates (i.e., splice junction protein coordinate will be 20.5 if an upstream CDS ends at 20 and the downstream begins at 21). For each transcript/proteome isoform, each peptide is then checked against the junction coordinates and is considered as junction-covering if it has at least one amino acid encoded on either side of a splice junction. A summary table is generated from the junction-covering peptides, which includes the number of junction-covering peptides generated by each enzyme, the number of total and unique junctions that a digest scheme covers (to avoid double counting of splice junctions shared between transcripts), and junction coverage percentage, which is the percentage of the total junctions in the transcript isoforms that are covered by digested peptides. An example junction summary table is in Table S4.

Isoform-unique peptides can be used to identify a protein isoform and to discriminate between protein/transcript variants with different functions. The isoform-unique peptides come from exons that cover unique regions (1) due to alternative splicing events and (2) N or C termini of the proteins resulting from different translation start or termination sites. ProtView also provides the number of isoform-unique peptides in each specific digest or set of filtered peptides (e.g., junction-covering), which can be appended onto either of the summary tables generated.

### Datasets

First, the Araport11 *A. thaliana* proteome^26^ is used in this manuscript as an example to illustrate the range of analyses that can be performed by ProtView and related information that can be generated. The Arabidopsis protein sequence database and its corresponding GFF3 files were downloaded from the TAIR database (https://www.arabidopsis.org/download/index-auto.jsp?dir=%2Fdownload_files%2FSequences%2FAraport11_blastsets and https://www.araport.org/data/araport11). The *A. thaliana* proteome was digested *in silico* by the Rapid Peptides Generator (RPG) with Arg-C, Asp-N(-UD), chymotrypsin (high specificity), Glu-C(-UD), Lys-C, Lys-N, trypsin, and pairwise combinations of these proteases in concurrent (represented in this article by ‘-’) and parallel digests (represented in this article by ‘/’). These proteases were chosen because they are highly specific, common alternative proteases and often paired with trypsin in the literature.

We have also compared results to a number of published proteomics studies that carried out different enzymatic digestions to validate the *in silico* analysis results of ProtView. Choudhary et al. (2003)^15^ examined a human recombinant tissue plasminogen activator protein using trypsin, Lys-C, Asp-N, and their parallel combinations, reporting protein sequence coverage % for each digest scheme. Swaney et al. (2010)^5^ examined *S. cerevisiae* digests with trypsin, Arg-C, Asp-N, Lys-C, Glu-C, and all these proteases in parallel, also reporting protein sequence % for each digest scheme. Guo et al. (2014)^14^ used trypsin, chymotrypsin, elastase, Glu-C, Lys-C, Asp-N, and Arg-C to map the HeLa proteome, reporting mean sequence coverage % for each digest scheme. The proteins and digest schemes used in the publications were used in ProtView to generate theoretical protein sequence coverage percentages for each digest scheme. The proteomes and protein sequences under examination in these publications were downloaded and digested in silico. The Human recombinant DNA derived tissue plasminogen activator protein sequence, the *S. cerevisiae* proteome, and the human proteome were downloaded from Uniprot (https://www.uniprot.org/uniprot/P00750, https://www.uniprot.org/proteomes/UP000002311, https://www.uniprot.org/proteomes/UP000005640) and digested using the same digest schemes as in the publications. Peptide identifications obtained by Guo et al. (2014)^14^ were downloaded from PRIDE and further methodology on the analysis and comparisons of these peptides can be found in the Supporting Information. All *in silico* digests were carried out using the Rapid Peptides Generator (RPG)^21^ and filtered with ProtView for a recommended length of 7–35 amino acids. Protein sequence coverage values were then compared between those obtained experimentally from these publications and the ones predicted by ProtView.

## Results and Discussion

### Using ProtView to Analyze Digests in a Proteomic Context

#### Selecting Enzymes with the Highest Protein Sequence Coverage

The purpose of the ProtView tool is to guide the choice of protease in *in vitro* experiments, by providing useful statistics on peptides generated *in silico*. One of these measurements is the percentage of the original protein sequence that is covered by the digested and potentially identifiable peptides. The coverage values given by ProtView are the theoretical upper limit, which is expected to be higher than those obtained in the laboratory. However, ProtView is useful in being able to show how digest schemes perform relative to one another.

Due to the number of possible combinations, Table 3 only includes trypsin in concurrent and parallel combination with Asp-N, chymotrypsin, and Lys-C. Results for the remaining combinations can be found in the Table S5. Summary statistics in Table 3 include the total number of peptides before and after filtering by length (7–35 aa length used here), mean lengths of the peptides generated, the number of isoform-unique peptides, and sequence coverage %. It is not surprising that the total numbers of digested peptides both pre- and post-filtering are higher for protease combinations than they are for single proteases due to combining multiple sets of peptides in the case of parallel digests (‘/’) or increased cleavage sites by using multiple proteases in concurrent digests (‘-’). For example, combining chymotrypsin with trypsin, up to 18.5% of protein coverage increase is predicted compared with trypsin digestion alone. Despite generating a high number of unfiltered peptides, the concurrent trypsin-Asp-N and trypsin-chymotrypsin combinations give relatively low sequence coverage % in comparison to the other digests due to many digested peptides being shorter than 7 amino acids and therefore below the filtering threshold. The concurrent trypsin-Lys-C combination gives a slight increase in sequence coverage (0.1%) when compared to the single tryptic digest, likely due to Lys-C cleaving after proline, whereas trypsin alone does not^4^ and therefore increasing cleavage frequency. This combination is favored *in vitro* because while both proteases cleave at Lys, Lys-C is more efficient at Lys cleavage than trypsin, and therefore combining them reduces the number of miscleaved peptides.^17^ It should be noted that the order in which the enzymes are added to a sample concurrently does not affect the results *in silico*; however, this may not be the case *in vitro*.

**Table 3:** Summary Statistics of *In Silico* Digests Carried out on *A. thaliana*, with Concurrent Protease Combinations Separated by ‘-‘ and Parallel by ‘/’, Sorted from Highest to Lowest Protein Sequence Coverage %, Including Residue Coverage Statistics Using Cys (C), Ser (S), and Lys (K)

enzyme	total peptides	mean length	filtered peptides (7–35 aa)	protein sequence coverage (%)	C coverage	S coverage	K coverage
trypsin/chymotrypsin	4,053,121	10.3	1,889,594	88	91.9	88.9	78.6
trypsin/Asp-N	3,550,802	11.7	1,679,339	85	88.1	86.3	74.8
trypsin/Lys-C	3,017,079	13.8	1,432,830	77.9	82.1	79	59.1
trypsin-Lys-C	2,455,307	8.5	1,042,209	69.6	75.3	73.2	42.3
trypsin	2,377,488	8.8	1,028,635	69.5	75.1	72.7	44.6
chymotrypsin	1,675,637	12.4	878,560	64.1	68.6	63.4	63.4
trypsin-Asp-N	3,395,134	6.1	1,093,276	63.4	71.5	70.7	33.8
trypsin-chymotrypsin	4,015,741	5.2	1,078,818	59.2	66	67.6	28.7
Lys-C	1,377,666	15.1	719,763	56	58.1	54.9	52.3
Lys-N	1,380,762	15.1	719,633	56	58.1	59.9	52.2
Glu-C	1,422,935	14.7	712,473	55.1	55.1	56. 2	54.1
Asp-N	1,180,854	17.7	653,902	52.9	53.8	51.9	55
Arg-C	1,173,294	17.8	620,534	50.4	51.5	50.1	51.1

#### Selecting Enzymes with the Highest Coverage of Specific Residues

Amino acid composition differs across protein types and families, and the level of post-translational modification differs between amino acids. For example, Lys and Arg, the residues that trypsin cleaves at, are enriched 2.37-fold and 1.95-fold at exon–exon junctions^7^ but are less frequent in membrane proteins.^6^ In eukaryotes, acetylation, methylation, ubiquitination, SUMOylation, and NEDDylation are a few of the enzymatic PTMs that occur on Lys residues.^27,28^ S-Acylation, prenylation, and phosphorylation all occur enzymatically on cysteines (Cys),^29,30^ alongside a range of reversible (nitrosylation, glutathionylation, disulfide, sulfenylation) and irreversible (sulfiniylation, sulfonylation) cysteine oxidation states,^31^ while serine (Ser) and threonine (Thr) residues are the best characterized sites of phosphorylation.^32^ Cys, Ser, and Lys also provide the nucleophile in many enzyme active sites^33,34^ and are often the targets of drugs, toxins, or activity probes. Being able to plan a digest around maximizing the coverage of a specific residue may therefore prove useful in the context of specific biological questions. Options to filter for peptides containing a specific amino acid (or motif) and calculating amino acid (or motif) coverage are included in ProtView, which is shown as the percentage of an amino acid in the original sequence that is covered by peptides after filtering for length.

Table 3 shows residue coverage of Cys (C), Ser (S), and Lys (K) from *in silico A. thaliana* digests to exemplify how much residue coverage % can differ between digest schemes. The digest scheme that gives the highest Lys coverage *in silico* is trypsin/chymotrypsin (78.6%) followed by trypsin/Asp-N (74.8%), with chymotrypsin giving the highest Lys coverage out of the single protease digests (63.4%). This would suggest that these digests may be favorable to use in analyses focused on the study of PTMs associated with Lys, although various PTMs block lysine cleavage and biological knowledge should be applied when interpreting the data. For all three of the residues (Cys, Ser, Lys) examined here, parallel protease combinations give the highest coverages, with the exception of trypsin/Lys-C giving lower Lys coverage (59.1%) due to both of these proteases cleaving at Lys. It should be noted that the protease that gives the highest residue coverage *in silico* may be otherwise unsuitable for use in a certain analysis or require adaptations to the experimental design *in vitro*. For example, in the context of ubiquitination, Glu-C digestion will result in peptides with a STLHLVLRLRGG ubiquitin remnant attached to Lys, causing a +1302.79 Da mass shift that needs to be taken into account in the database search.^35^ As mentioned, various PTMs will block proteolytic cleavage from taking place at a specified residue, so the nature of the modification needs to be taken into account before examining theoretical coverage *in silico*.

### Using ProtView to Predict Digest Outcomes in a Splice-Variant-Focused Transcriptomic Context

ProtView provides unique capabilities to examine transcriptomic regulations using proteomic evidence by mapping the digested peptides to the genome reference. ProtView identifies junction-covering peptides and provides junction summary information for each digest scheme. This information is shown in Table 4, consisting of the number of junction-covering peptides, number of junctions covered, unique junctions covered, and the percentage of junctions that are covered by peptides from each digest after filtering. The number of isoform-unique peptides was calculated for both the entire sets of peptides generated and the junction-covering peptides.

**Table 4:** Junction Summary Statistics Generated for *A. thaliana* Using ProtView, with Concurrent Protease Combinations Separated by ‘-‘ and Parallel by ‘/’

enzyme	junction spanning peptides	unique junctions covered	total junctions covered	total junction coverage (%)	isoform-unique peptides	junction covering isoform-unique peptides
trypsin/chymotrypsin	286,442	101,973	201,015	84.5	545,769	65,589
trypsin/Asp-N	252,463	93,752	184,591	77.5	473,080	57,837
trypsin/Lys-C	207,385	80,087	156,990	66	408,117	48,464
chymotrypsin	152,914	77,761	152,914	64.2	258,574	34,697
trypsin	133,528	68,070	133,528	56.1	287,199	30,892
trypsin-Lys-C	133,484	68,079	133,484	56.1	290,657	30,917
Lys-N	129,305	65,925	129,305	54.3	207,805	29,634
trypsin-Asp-N	121,400	61,776	121,400	51	299,274	27,788
Asp-N	119,183	60,460	119,183	50.1	186,917	27,022
Glu-C	117,824	59,630	117,824	49.5	198,510	26,770
trypsin-chymotrypsin	114,895	58,311	114,895	48.3	291,116	26,014
Lys-C	111,835	57,631	111,835	47	207,580	26,656
Arg-C	106,708	54,338	106,708	44.8	182,158	246,73

Table 4 exemplifies the format of a junction summary table generated by ProtView, with the digest schemes sorted in the order from highest to lowest junction coverage. The output shows that in terms of single enzyme digests, chymotrypsin outperforms trypsin in terms of the number of junction-covering peptides generated and junctions covered for the Arabidopsis proteome, further underlining the point that trypsin may not always be the most optimal choice depending on the focus of the investigation. In addition, chymotryptic cleavage *in silico* should provide a closer match for what can be expected *in vitro* due to not being blocked by PTMs. If carrying out an analysis where maximizing splice junction coverage is a priority, these results suggest that combining chymotryptic peptides in parallel with tryptic peptides can theoretically give a 28.4% increase in junction coverage compared to trypsin alone, in addition to generating the most isoform-unique peptides that can be used to discriminate between protein isoforms.

**Figure 3:**
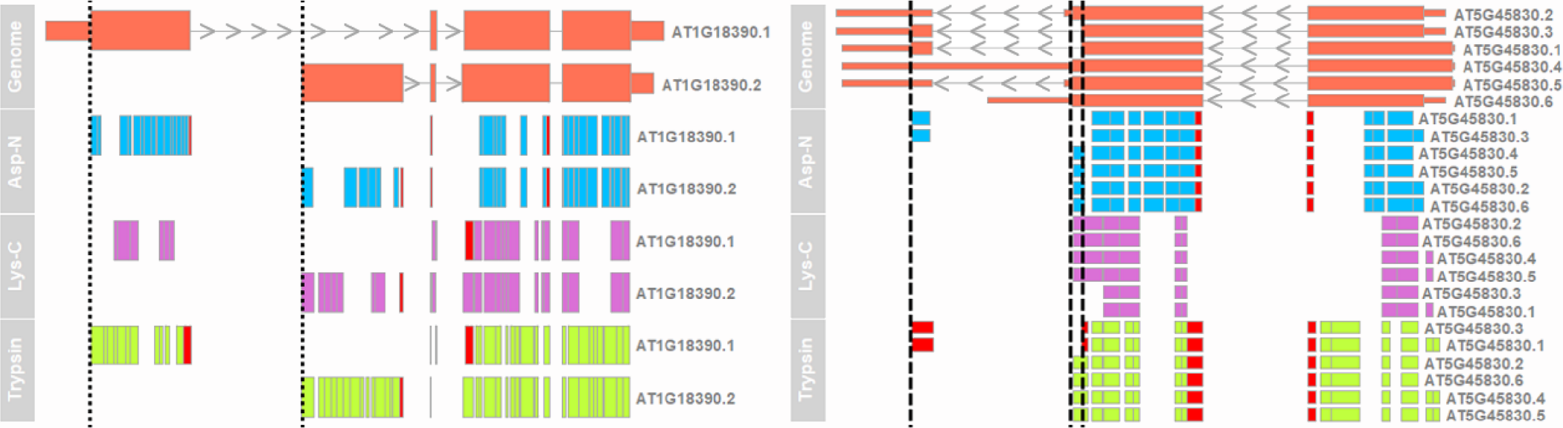
AT1G18390 and AT5G45830 *in silico* generated peptides mapped onto the genome. The first row shows the genome; thick colored boxes represent exons, thin colored boxes represent UTRs, gray lines linking between exons represent introns, and vertical lines show alternative translational start (AT1G18390) (dotted) or stop (AT5G45830) (dashed) sites. The subsequent rows represent peptides mapped onto the genome, with exon–exon junction-covering peptides in red.

In addition, ProtView allows the downstream examination and visualization of digested peptides to identify post-transcriptional regulations. For example, AT1G18390 is a gene with two transcript/protein isoforms with alternative transcription start sites. Visualization of AT1G18390 *in silico* generated peptides on the genome (Figure 3) shows coverage of the alternative transcriptional start sites (vertical dashed lines) by peptides. It allows isoform-specific and exon–exon junction covering peptides to easily be identified. In this example, trypsin and Asp-N generate peptides that cover the transcription start sites in both isoforms, while the peptides generated by Lys-C do not cover the translational start site of the AT1G18390.1 isoform. We can also see that the predicted regions with proteomics data coverage for the first exon of AT1G18390.1 are highly complementary between Asp-N and trypsin. Similarly, AT5G45830 is a gene on the negative strand with alternative stop sites due to polyadenylation events. Lys-C digestion also fails to capture the translation stop site (Figure 3) and is thus not suitable for tasks where experimental evidence for that region is required. Protview provides great resolution information between the regions of transcripts and the potential proteomic evidence, allowing transcriptional regulation to be examined using proteomic data. The analysis also highlights the usefulness of additional enzymatic digestion in specific regions, thus providing guidance and improving the efficiency of follow-up experiments.

### Identification of Inaccurate Digestion Rules

During comparison of ProtView prediction capabilities against *in vitro-*generated proteomic data (results discussed in full in the subsequent section), we observed a number of anomalies in enzyme coverage rankings with regard to Asp-N and Glu-C. The RPG cleavage rules for Asp-N include cleavage at all Cys, despite Asp-N actually cleaving at cysteic acid, a vanishingly rare oxidation form of Cys in natural protein samples,^36^ which results in over inflated protein coverage predicted by ProtView (67.1%) for the Choudhary et al. dataset. The regions covered by Choudhary et al., as estimated from figure 5 in their manuscript depicting sequence coverage by different enzymes, do not show cleavage before Cys by Asp-N. After revising the cleavage rule to only cleave before Asp, the ProtView protein coverage is dropped to 40.2%, comparable to the protein coverage of 34.9% determined by experimental data by Choudhary et al. (2003). In addition, to study the cleavage of Asp-N in vitro, we subsequently downloaded identified peptide sequences generated by Asp-N digestion from Guo et al. (2014). By analyzing the identified peptides, we found that only 9 of the 10,316 assigned peptide sequences from CID analysis had an N-terminal Cys residue, while 9072 were generated by cleavage at Asp and 1235 by cleavage at another residue. Similarly, only 6 of the 12,292 peptide sequences from HCD analysis for Asp-N contained an N-terminal Cys, while 10,718 were generated by cleavage at Asp and 1530 by cleavage at another residue. This data thus supports our proposed revised Asp-N digestion rule, which only cleaves at Asp.

Similarly, the original cleavage rule in RPG for Glu-C is after Asp or Glu. Glu-C cleavage specificity is reported to be condition-dependent, cleaving C-terminal to glutamyl bonds in ammonium bicarbonate and ammonium acetate buffers, or at both glutamic and aspartic residues in phosphate buffers. An examination of the peptides identified by Guo et al. (2014) using 100 mM Tris pH 8.0 for Glu-C revealed that 4862 of the CID derived peptide sequences end in Glu, while only 125 had a C-terminal Asp and 193 were generated by cleavage at other residues. HCD was similar, with 5286 of 5685 peptides having a C-terminal Glu, only 181 having a C-terminal Asp, and 218 having another amino acid at their C-terminus. We thus revised the Glu-C cleavage rule to only cleave after Glu and better represent the most common experimental conditions. This caused the ProtView coverage using Glu-C to drop from 65.5% to 53.4% in the Swaney et al. (2010) study, making the relative order and correlation of predicted and experimental coverages between digest schemes correct. Our user-defined versions of these proteases cleaving only at Asp (Asp-N-UD) and Glu (Glu-C-UD) are included in ProtView installation and were used in all analyses/comparisons in this study. The options for either version of each protease remain available and grant the user the flexibility to select appropriately based on the conditions of their experiment.

### Comparing ProtView *In Silico* Results with *In Vitro* Experimental Data

Following cleavage rule revision based on *in vitro* data, we compared ProtView’s predictive ability against several published multiprotease data sets. In the first study, comparing sequence coverage % obtained by Choudhary et al. (2003) on human recombinant protein to the coverage % predicted by ProtView (Table 5), the highest sequence coverage is obtained by using parallel enzyme digests, with trypsin/Lys-C/Asp-N (93.3% for Choudhary et al., 93.4% for ProtView) followed by trypsin/Lys-C (88.2% and 91.8%), trypsin (65% and 85%), Lys-C (62.8% and 40.6%), and Asp-N (34.9% and 40.2%). Although the accuracy of the ProtView-predicted values for each digestion scheme varies, the relative performance and ranking of the digestion schemes are correctly predicted by ProtView.

**Table 5:** Experimental vs *In Silico-*Predicted Sequence Coverage % between Choudhary et al. (2003) and ProtView for Human Recombinant Tissue Plasminogen Activator

enzyme	experimental coverage %	ProtView-predicted coverage %
trypsin/Lys-C/Asp-N	93.3	93.4
trypsin/Lys-C	88.2	91.8
trypsin	65.0	85.0
Lys-C	62.8	40.6
Asp-N	34.9	40.2

**Figure 4:**
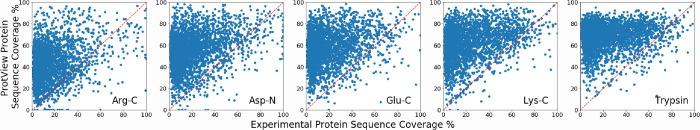
Sequence coverage comparisons for *S. cerevisiae* between Swaney et al. (2010) and ProtView for individual proteins digested by (i) Arg-C, (ii) Asp-N, (iii) Glu-C, (iv) Lys-C, and (v) trypsin.

In the comparison to Swaney et al. (2010), all enzymes in parallel achieved the highest sequence coverage (43.4% for Swaney et al., 93.4% for ProtView) followed by trypsin (24.5%, 68.9%), Lys-C (24.3%, 60.8%), Asp-N (21.5%, 54.7%), Glu-C (20.9%, 53.4%), and ending with Arg-C providing the lowest coverage (18.6%, 42.2%). The comparisons again shows that ProtView can correctly rank protease performances and provide rapid pre-analysis to assist in the choice of proteases for addressing a given experimental question (Table 6). We also generated predicted vs *in vitro* coverages obtained for individual proteins (Figure 4), highlighting that ProtView tends to calculate the theoretical upper limit of protein sequence coverage that could be obtained in an experiment. Instances where the coverage from the *in vitro* experiment is higher than that predicted by ProtView are likely due to larger (>35aa) peptides existing *in vitro* due to missed cleavage that were not considered *in silico* or smaller peptides that did not meet the ProtView peptide length filtering criteria (7–35 aa).

**Table 6:** Experimental vs *In Silico-*Predicted Sequence Coverage % between Swaney et al. (2010) and ProtView for the *S. cerevisiae* Proteome

enzyme	experimental coverage %	ProtView-predicted coverage %
all (in parallel)	43.4	93.4
trypsin	24.5	68.9
Lys-C	24.3	60.8
Asp-N	21.5	54.7
Glu-C	20.9	53.4
Arg-C	18.6	42.2

**Figure 5:**
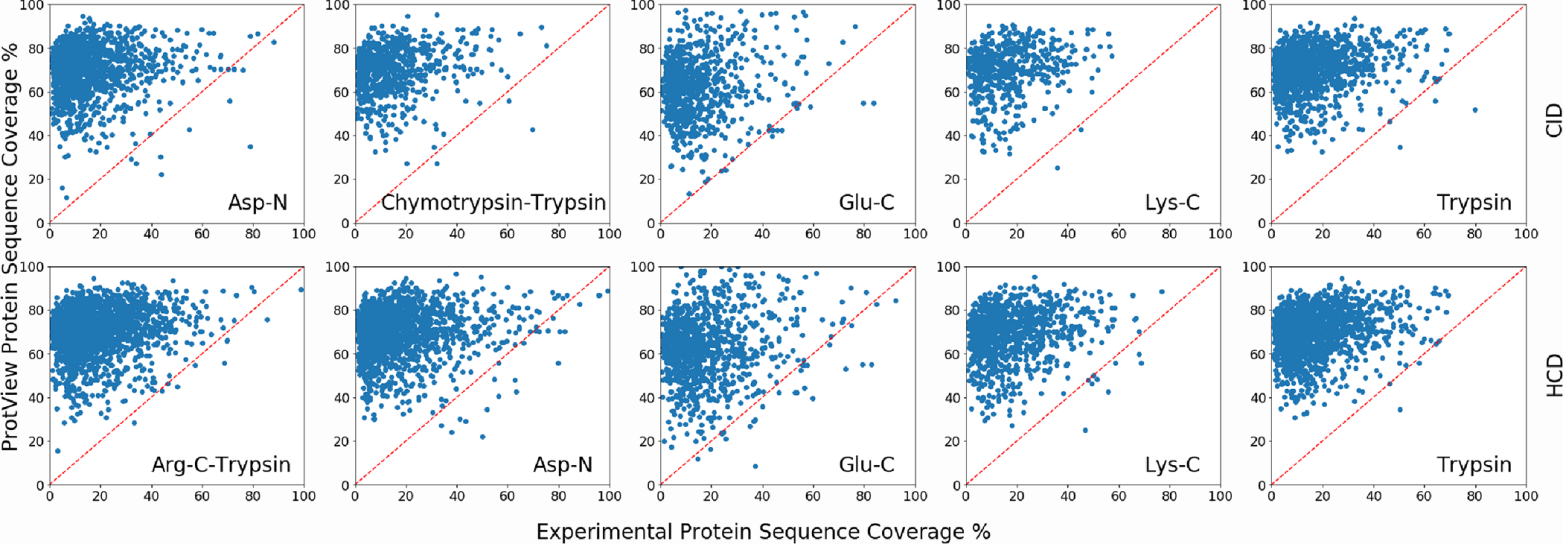
Sequence coverage comparisons for all human proteins with >3 peptide sequence matches in the Guo et al. (2014) database and ProtView CID (top row) and HCD (bottom row) fractionated digests using Asp-N, chymotrypsin-trypsin, Glu-C, Lys-C, and trypsin.

Finally, ProtView predictions were compared to CID and HCD fractionated digests from the confetti multiprotease HeLa database by Guo et al. (2014). Due to depth of protein coverage *in vitro* being largely dictated by fractionation capacity and sensitivity, Guo et al. (2014) went into a greater depth in their analysis on the five top-performing methods for SAX-fractionated digests by CID and HCD fragmentation. These digests were therefore selected for ProtView explorations. Mean sequence coverage of digest schemes from Table S4 of the publication were compared directly to ProtView-predicted upper limits of human proteome sequence coverage %. For CID fractionated digests, trypsin gave the highest mean coverage (24.8%) followed by chymotrypsin-trypsin (23.5%), Lys-C (17.9%), Glu-C (12.3%), and Asp-N (11.3%). ProtView protein sequence coverage values follow the same order, except for Glu-C, which may be due to the highly condition-specific nature of Glu-C digestion (trypsin: 67.0%, chymotrypsin-trypsin: 60.7%, Glu-C: 54.9%, Lys-C: 47.8%, Asp-N: 46.7%). For HCD fractionated digests, trypsin had the highest mean sequence coverage (26.9%) followed by Arg-C-Trypsin (23.2%), Lys-C (20.3%), Glu-C (14.7%), and Asp-N (13.6%). The protein sequence coverage values of individual proteins were compared to those predicted by ProtView, as shown in Figure 5, which similarly shows that ProtView tends to generate the upper limit of achievable protein sequence coverage % in most cases. In the predictions generated by ProtView, Arg-C-trypsin gives the highest protein sequence coverage by 0.5% (Arg-C-trypsin: 67.5%, trypsin: 67%). Given the minor difference between the ProtView-generated coverage values, the order of prediction being reversed *in vitro* for trypsin and chymotrypsin-trypsin is not of great concern; however, Glu-C is the outlier again. Glu-C accuracy has improved after our digestion rule revision, but given its condition-dependent nature, further study is required to deepen our understanding of its biochemistry and behavior and further improve prediction accuracy (Table 7).

**Table 7:** Experimental Mean Sequence Coverage % for CID and HCD Fractionated Digests vs *In Silico-*Predicted Sequence Coverage % between the Guo et al. (2014) Multiprotease HeLa Database and ProtView for the Human Proteome

protease	ProtView coverage %	mean CID coverage %	order	mean HCD coverage %	order
Arg-C-trypsin	67.5	n/a	n/a	23.2	2
trypsin	67	24.8	1	26.9	1
chymotrypsin-trypsin	60.7	23.5	2	n/a	n/a
Glu-C	54.9	12.3	4	14.7	4
Lys-C	47.8	17.9	3	20.3	3
Asp-N	46.7	11.3	5	13.6	5

**Figure 6:**
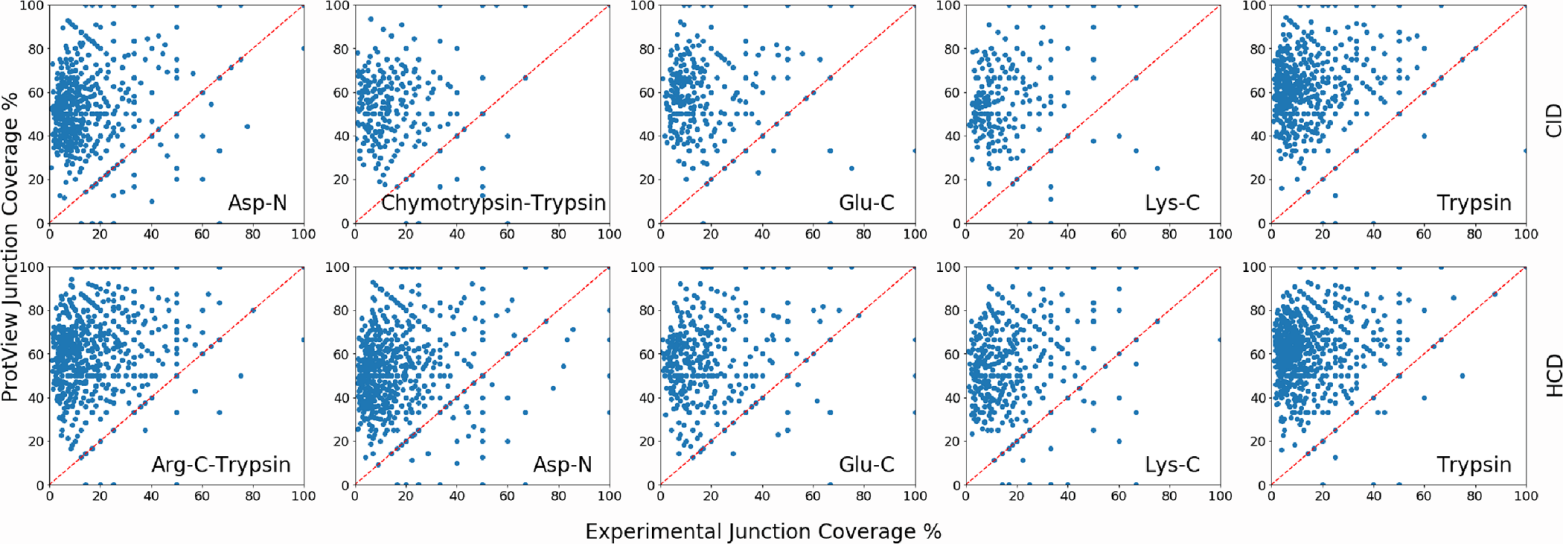
Junction coverage comparisons for human proteins with >3 peptide sequence matches in Guo et al. (2014) database and ProtView CID (top row) and HCD (bottom row) fractionated digests Asp-N, chymotrypsin-trypsin, Glu-C, Lys-C, and trypsin.

For the proteogenomic exploration, peptides identified in the Guo et al. (2014) Confetti database were downloaded from PRIDE and mapped to exon junctions in the genome. For proteins with >3 matched peptides in the HCD analyzed *in vitro* digests by Guo et al. (2014), Arg-C-Trypsin gives the highest number of junctions in the database covered by identified peptides (2236) followed by Trypsin (2158), Asp-N (1,941), Lys-C (1,108), and Glu-C (1,051). Again, the order predicted by ProtView matches most of the experimental results, except for Glu-C, which ranks lower than predicted (Arg-C-trypsin: 59%, trypsin: 58.8%, Glu-C: 51.4%, Asp-N: 45.8%, Lys-C: 44.7%) as shown in Table 8. Junction coverage comparison plots for individual proteins with >3 peptide matches show ProtView giving the upper limit of achievable junction coverage % for most proteins (Figure 6). For proteins with >3 matched peptides in the CID fractionated *in vitro* digests, trypsin gives the highest number of junctions in the human database covered by identified peptides (1482) followed by Asp-N (1,411), Glu-C (705), chymotrypsin-trypsin (622), and Lys-C (498). This time, the order predicted by ProtView matches the experimental results, except for Asp-N and Glu-C (trypsin: 58.8%, Glu-C: 51.4%, chymotrypsin-trypsin: 52.7%, Asp-N: 45.8%, Lys-C: 44.7%).

**Table 8:** Experimental Exon Junctions Covered by HCD and CID Fractionated Digests vs *In Silico-*Predicted Exon Junction Coverage % between the Guo et al. (2014) Multiprotease HeLa Database and ProtView for the Human Proteome

protease	ProtView junction coverage %	HCD junctions covered	HCD junctions covered order	CID junctions covered	CID junctions covered order
Arg-C-trypsin	59	2236	1	n/a	n/a
trypsin	58.8	2158	2	1482	1
chymotrypsin-trypsin	52.7	n/a	n/a	622	4
Glu-C	51.4	1051	5	705	3
Asp-N	45.8	1941	3	1411	2
Lys-C	44.7	1108	4	498	5

The comparisons carried out between ProtView and published proteomic data demonstrate the ability of ProtView to generate the theoretical upper limit of achievable protein sequence and exon junction coverage while being able to correctly evaluate proteases relative to one another in most cases. Using RNA-seq data to generate custom databases that are specific to the tissue, cell type, or developmental stage under examination could improve ProtView *in silico* prediction correlation with *in vitro* data, particularly when analyzing single cell types from multi-tissue organisms, where the transcriptional and post-transcriptional profile of the cell type under study may diverge greatly from the reference tissue, organism, or condition profile (e.g., cancer cell lines such as HeLa strain variant was used in the study). This partially explains the low proteome coverage in the CID and HCD experiments.

## Conclusions

Evaluation of digest schemes *in silico* can save on time and resources compared to *in vitro* evaluations. ProtView is a novel software tool for the evaluation of digest schemes, designed to process and analyze *in silico* digest output by different enzymes and their combinations in multiple contexts. It is clear from our analysis that, depending on the focus of the investigation, the ideal choice of enzyme could vary considerably. The enzyme combinations that provide the best protein sequence coverage do not necessarily provide the best view of the proteome in terms of specific PTMs; ProtView is therefore a timely, tailored experimental planning solution.

Preliminary validations show that ProtView can reliably predict the majority of experimentally determined protein sequence coverage orders between digest schemes. Our analysis also shows that one difficulty of *in silico* digestion lies in that the digestion rule often does not accurately reflect what happens in the experiment. Therefore, to judge how well a protease will perform also highly depends on the conditions that these digestions will be carried out in. Analysis of digestion products also helps us understand how the digest process affects the coverage of the proteome.

There is generally a lack of consistency and low correlations in terms of gene expression and corresponding protein abundances between transcriptomics experimental data and proteomic evidence. This is partly due to the lack of tools and methods that could map transcript and protein information in detail. ProtView is filling this gap. ProtView is the first tool that maps predicted peptides from digestion to the reference genome, which allows transcriptomic activities, such as transcriptional (alternative transcriptional start sites and stop sites) and post-transcriptional regulations (alternative splicing), to be studied using proteomic experimental evidence. Mapping peptides to the genome also creates the possibility in the future to integrate sequence variations of different species and their subspecies (e.g., cultivars/eco-types/landraces in plants), to derive a list of individualized peptides that could possibly be detected in mass spectrometry-based proteomics experiments.

## Supporting Information Available

Supplementary methods: conversion equations between relative proteomic and genomic coordinates, parameters for comparisons to Guo et al. (2014) database; Table S1: cleavage specificities of the enzymes used in RPG digests in the benchmark analyses in this article; Table S2: example of a digested peptide processed by ProtView; Table S3: format of CDS after processing and extraction from gff3 files; Table S4: example junction summary statistics of a digested protein; Table S5: summary statistics of *in silico* digests carried out on *A. thaliana* for all proteases and pairwise protease combinations; Table S6: junction summary statistics generated for *A. thaliana* using ProtView for all proteases and pairwise protease combinations; Figure S1: illustration example of relative peptide coordinates and their corresponding coordinates on the genome (PDF)
