# Supplementary material for: ProtView: A Versatile Tool for In Silico Protease Evaluation and Selection in a Proteomic and Proteogenomic Context
Source: J Proteome Res. 2023 May 29;22(7):2400–10. doi: 10.1021/acs.jproteome.3c00135 (PMC10337256; doi:10.1021/acs.jproteome.3c00135)
Supplement: Supplementary file 1 — pr3c00135_si_001.pdf [file pr3c00135_si_001.pdf]

Supporting Information

ProtView: A versatile tool for *in silico* protease evaluation and selection in a proteomic and proteogenomic context

*Sophia S. Puliasis[1,2], Dominika Lewandowska[3], Piers Hemsley[1,3], Runxuan Zhang[2]**

[1]Division of Plant Sciences, School of Life Sciences, University of Dundee, Dow Street, Dundee, DD1 5EH, Scotland, UK

[2]Information and Computational Sciences, The James Hutton Institute, Invergowrie, Dundee, DD2 5DA, Scotland, UK

[3]Cell and Molecular Sciences, The James Hutton Institute, Invergowrie, Dundee, DD2 5DA, Scotland, UK

*Correspondence to Dr Runxuan Zhang

Email: Runxuan.Zhang@hutton.ac.uk

Name	Caption
Supplementary Methods	Additional methodology, including conversion equations between relative proteomic and genomic coordinates and comparisons to Guo et al (2014)
Supplementary Table 1	Cleavage specificities of the enzymes used in RPG digests in the benchmark analyses in this article
Supplementary Table 2	Example of a digested peptide processed by ProtView
Supplementary Table 3	Format of CDS after processing and extraction from gff3 files
Supplementary Table 4	Example junction summary statistics of a digested protein

Supplementary Table 5	Summary statistics of in silico digests carried out on A. thaliana for all proteases and pairwise protease combinations ('/' for parallel, '-' for concurrent)
Supplementary Table 6	Junction Summary statistics generated for A. thaliana using ProtView for all proteases and pairwise protease combinations ('/' for parallel, '-' for concurrent)
Supplementary Figure 1	Illustration example of relative peptide coordinates and their corresponding coordinates on the genome

Supplementary Methods

Genomic coordinate conversions

CDS and intron IDs consist of chromosome, start position, end position, and strand (e.g., Chr1_24848737_24848859_+). While converting CDS genomic coordinates to their relative proteomic coordinates, intron lengths between CDS are added up separately for each isoform, to give cumulative intron length of the introns preceding each CDS within an isoform. To obtain the relative protein coordinates for a CDS on the positive strand, the translation start position of the gene and cumulative intron length (after translation start position) are subtracted from the CDS genomic coordinate increased by one, then divided by 3 to take the triplet code into account. The final number is rounded up to the nearest integer as shown in the equation below.

While for CDS on the negative strand, the same equation can be applied with adjustments shown below.

$$relative\ protein\ coordinate\ (positive\ strand) = RoundUp(\frac{(coordinate - translation\ start\ position\ of\ gene + cumulative\ intron\ length + 1)}{3})$$

$$relative\ protein\ coordinate\ (negative\ strand) = RoundUp(|\frac{(coordinate - translation\ start\ position\ of\ gene + cumulative\ intron\ length + 1)}{3}|)$$

Output from this step is saved as two csv files, one for each DNA strand, and allows for the downstream conversion of relative proteomic peptide coordinates to genomic and the identification of peptides covering splice junctions.

The following equations are used by ProtView to convert relative protein coordinates to their corresponding genomic coordinates:

$$genomic\ start\ position\ (positive\ strand) = translation\ start\ coordinate\ + cumulative\ intron\ length + 3 * (protein\ coordinate - 1)$$

$$genomic\ end\ position\ (positive\ strand) = translation\ start\ coordinate\ + cumulative\ intron\ length + 3 * (protein\ coordinate - 1) + 2$$

$$genomic\ start\ position\ (negative\ strand) = translation\ start\ coordinate - cumulative\ intron\ length - 3 * (protein\ coordinate - 1) - 2$$

$$genomic\ end\ position\ (negative\ strand) = translation\ start\ coordinate - cumulative\ intron\ length - 3 * (protein\ coordinate - 1)$$

Comparisons to peptides identified by Guo et al. (2014)

Proteomic comparisons

Canonical protein sequences from UniProt were digested in-silico with Arg-C-Trypsin, Asp-N, Chymotrypsin-Trypsin, Glu-C, and Lys-C to match the fractionated digests in the confetti analysis. The generated peptides were filtered for length 7-35 aa and sequence coverage % was calculated for each protein/isoform in the dataset. Sequence coverage % generated by ProtView was compared to mean sequence coverage % values reported in supplementary table 4 from Guo et al. (2014).

Proteogenomic comparisons

The browser extensible data (BED) file from the UniProt database was filtered for accessions that had only one annotation and corresponded to isoform sequences in the fasta database, converted to gff3 format, and processed with ProtView to identify exon junction coordinates. ProtView was used to map the peptides generated in silico

to exon junctions. An in-house script based on the ProtView function was used to map peptides identified by Guo et al (2014) to exon junctions for proteins/isoforms with >3 mapped peptides.

Supplementary Table S1. Example of a digested peptide after processing digest results. The table contains information on the enzyme used in the theoretical digest, relative peptide start and cleavage position (end) on the protein sequence, size in amino acids, molecular weight, isoelectric point, peptide sequence, and the protein that the peptide originates from

enzyme	Peptide start	Peptide End	Peptide size	Mol weight	Isoelectric point	Sequence	Parent
Trypsin	1	8	8	991.1297	7.77	MFSNIDHK	AT1G66600.1

Supplementary Table S2. Format of CDSs after processing and extraction from GFF3 files

type	start	end	strand	Parent	CDS ID	Intron ID	Intron length	Cumulative intron length	Protein start	Protein end
CDS	24848737	24848859	+	AT1G66600.1	Chr1_24848737_24848859_+	Chr1_24848651_24848736_+	86	86	83	123

Supplementary Table S3. Junction summary statistics of digested proteins AT1G66600 and AT1G66610

Enzyme	Junction spanning peptides	Unique junctions covered	Total junctions covered	Total junction coverage (%)
Asp-N	2	2	2	33.33333
Glu-C	6	5	6	100

Supplementary Table S4. Cleavage specificities of the enzymes used in RPG digests in the benchmark analyses in this article

Residue	R		C		D		F		Y		W		E		K		Exceptions
Terminal	C	N	C	N	C	N	C	N	C	N	C	N	C	N	C	N	

Enzyme																	
ArgC	X																
Asp-N (RPG)				X		X											
Asp-N						X											
Chymotrypsin (high specificity)							X		X		X						FP, YP, WP, WM
Glu-C (RPG)					X								X				
Glu-C													X				
Lys-C															X		
LysN																X	
Trypsin	X														X		KP, RP, CKD, DKD, CKH, CKY, CRK, RRH, RRR

Supplementary Table S5. Summary statistics of in silico digests carried out on A. thaliana for all proteases and pairwise protease combinations (‘/’ for parallel, ‘-’ for concurrent)

**Enzyme**	**Total Peptides**	**Mean Length**	**Mediam Length**	**Filtered Peptides**	**Sequence Coverage %**	**Isoform Unique Peptides**
Arg-C	1173294	17.77540753	11	620534	50.405588	182158
Arg-C/Asp-N-UD	2349939	17.7500599	11	1272241	76.296412	368227
Arg-C/Chymotrypsin-high	2848912	14.64122374	9	1499091	81.850263	440727

Arg-C/GluC-UD	2596186	16.06647521	10	1332994	77.045724	78.20201592
Arg-C/Lys-C	2550939	16.35145254	10	1340291	77.774606	389723
Arg-C/Lys-N	2547448	16.37386043	10	1337483	77.773959	388979
Arg-C/Trypsin	3009921	13.85802418	7	1414582	78.677555	401303
Arg-C-Asp-N-UD	2246239	9.284755095	6	1056106	71.131464	295885
Arg-C-Chymotrypsin-high	2800617	7.446851533	5	1104433	69.322119	308969
Arg-C-GluC-UD	2547909	8.185448931	5	1033774	68.319318	74.40246582

Arg-C-Lys-C	2502642	8.333504752	5	1050942	69.671092	292816
Arg-C-Lys-N	2424059	8.603659812	6	1050942	69.671092	294907
Arg-C-Trypsin	2435752	8.562357334	6	1054844	69.924072	289667
Asp-N-UD	1180854	17.66160677	12	653902	52.926059	186917
Asp-N-UD/Chymotrypsin-high	2849772	14.63680533	9	1529210	82.879033	444359
Asp-N-UD/GluC-UD	2596863	16.06228669	10	1363421	77.307388	78.54402518
Asp-N-UD/Lys-C	2553863	16.33273124	10	1371440	78.407802	393678

Asp-N-UD/Lys-N	2561591	16.28345743	10	1373524	78.423295	394710
Asp-N-UD/Trypsin	3550802	11.7470808	7	1679339	85.016283	473080
Asp-N-UD-Chymotrypsin-high	2711909	7.690442047	5	1119792	70.391559	311215
Asp-N-UD-GluC-UD	2466065	8.457108389	6	1044433	68.06511	74.18668049
Asp-N-UD-Lys-C	2438657	8.552157601	6	1059013	69.445423	295234
Asp-N-UD-Lys-N	2513298	8.298171964	5	1056094	69.202177	294772
Chymotrypsin-high	1675637	12.44647797	8	878560	64.083725	258574

Chymotrypsin-high/Lys-C	3053296	13.66115765	9	1598319	84.324076	466150
Chymotrypsin-high/Lys-N	3050069	13.67561127	9	1595540	84.321343	465440
Chymotrypsin-high-GluC-UD	3050252	6.837395402	5	1129444	68.497753	74.51559599
Chymotrypsin-high-Lys-C	3004985	6.940393712	5	1120839	68.325494	309886
Chymotrypsin-high-Lys-N	2909465	7.168252239	5	1128967	68.769678	311870
GluC-UD	1422935	14.656874	9	712473	55.120046	56.19872152
GluC-UD/Chymotrypsin-high	3098560	13.46159442	8	1591029	84.199344	86.74203441

GluC-UD/Lys-C	2800558	14.89401684	9	1432219	78.494325	80.07940062
GluC-UD/Lys-N	2794874	14.92430714	9	1428861	78.490863	80.09799377
GluC-UD/Trypsin	3800413	10.97553292	7	1741104	85.659706	88.43977259
GluC-UD-Lys-C	2752280	7.577637086	5	1009527	65.778147	73.84833748
GluC-UD-Lys-N	2643368	7.889850751	5	1013066	66.060515	73.69095037
GluC-UD-Trypsin	3763032	5.542280533	4	1034383	60.448526	70.63303139
Lys-C	1377666	15.13848712	9	719763	56.03467	207580

Lys-C/Lys-N	2744574	15.19782596	10	1439365	57.700587	415356
Lys-C-Lys-N	2598568	8.025873866	1	665792	52.145978	194045
Lys-N	1380762	15.104543	9	719633	56.043694	207805
Lys-N/Trypsin	3735893	11.16508369	7	1747316	79.453263	494614
Lys-N-Trypsin	3534951	5.89987782	2	976235	65.427165	274177
Trypsin	2377488	8.772191069	6	1028635	69.471124	287199
Trypsin/Chymotrypsin-high	4053121	10.29121953	7	1889594	87.956609	545769

Trypsin/Lys-C	3017079	13.82514611	7	1432830	77.943912	408117
Trypsin-Asp-N-UD	3395134	6.142844141	4	1093276	63.414759	299274
Trypsin-Chymotrypsin-high	4015741	5.193507002	4	1078818	59.224237	291116
Trypsin-Lys-C	2455307	8.494163459	6	1042209	69.581395	290657

Supplementary Table S6. Junction Summary statistics generated for A. thaliana using ProtView for all proteases and pairwise protease combinations ('/' for parallel, '-' for concurrent)

Enzyme	Junction Spanning Peptides	Unique Junctions Covered	Total Junctions Covered	Total Junction Coverage %	Isoform Unique Peptides
Arg-C	106708	54338	106708	44.83604766	182158
Arg-C/Asp-N-UD	225698	87015	171344	71.9944873	368227
Arg-C/Chymotrypsin-high	259622	96985	191119	80.30345048	440727
Arg-C/GluC-UD	224532	86736	171152	71.91381368	380653
Arg-C/Lys-C	218543	86269	169321	71.14447302	389723
Arg-C/Lys-N	235754	90064	177340	74.51385738	388979
Arg-C/Trypsin	211060	83177	163743	68.80073615	401303
Arg-C-Asp-N-UD	153573	77792	153573	64.52755508	368227
Arg-C-Chymotrypsin-high	154947	78335	154947	65.10487571	308969
Arg-C-GluC-UD	138729	70504	138729	58.29047547	287539

Arg-C-Lys-C	133636	68140	133636	56.15052354	292816
Arg-C-Lys-N	149667	75662	149667	62.88635103	294907
Arg-C-Trypsin	133794	68187	133794	56.21691121	289667
Asp-N-UD	119183	60460	119183	50.0777324	186917
Asp-N-UD/Chymotrypsin-high	271746	99016	195231	82.03121061	444359
Asp-N-UD/GluC-UD	236698	88887	175820	73.87519118	384397
Asp-N-UD/Lys-C	230799	88296	173411	72.86298929	393678
Asp-N-UD/Lys-N	248488	92315	181870	76.41725071	394710
Asp-N-UD/Trypsin	252463	93752	184591	77.56054724	473080
Asp-N-UD-Chymotrypsin-high	161502	81399	161502	67.85912368	311215
Asp-N-UD-GluC-UD	145656	73777	145656	61.20102859	287384
Asp-N-UD-Lys-C	140196	71414	140196	58.90687238	295234
Asp-N-UD-Lys-N	152967	77450	152967	64.27292896	294772
Chymotrypsin-high	152914	77761	152914	64.25065967	258574

Chymotrypsin-high/Lys-C	264749	98326	193618	81.35346813	466150
Chymotrypsin-high/Lys-N	281926	101082	199347	83.76065144	465440
Chymotrypsin-high-GluC-UD	147645	74743	147645	62.03675692	312116
Chymotrypsin-high-Lys-C	140548	71165	140548	59.05477403	309886
Chymotrypsin-high-Lys-N	153581	77369	153581	64.53091649	311870
GluC-UD	117824	59630	117824	49.5067144	198510
GluC-UD/Chymotrypsin-high	270738	99544	196502	82.5652532	457080
GluC-UD/Lys-C	229659	88457	174080	73.14408646	406066
GluC-UD/Lys-N	246754	91395	180193	75.71261702	405220
GluC-UD/Trypsin	251352	95517	188518	79.21057497	485696
GluC-UD-Lys-C	123820	63239	123820	52.02608447	279772
GluC-UD-Lys-N	137885	69874	137885	57.93584766	280687
GluC-UD-Trypsin	106158	54432	106158	44.60495134	283348
Lys-C	111835	57631	111835	46.99028555	207580

Lys-C/Lys-N	241140	68533	134210	56.39170406	415356
Lys-C-Lys-N	106737	55000	106737	44.84823274	194045
Lys-N	129305	65925	129305	54.33074505	207805
Lys-N/Trypsin	262789	89875	177200	74.45503286	494614
Lys-N-Trypsin	127429	64907	127429	53.54249651	274177
Trypsin	133528	68070	133528	56.10514462	287199
Trypsin/Chymotrypsin-high	286442	101973	201015	84.46150355	545769
Trypsin/Lys-C	207385	80087	156990	65.9632935	408117
Trypsin-Asp-N-UD	121400	61776	121400	51.00926066	299274
Trypsin-Chymotrypsin-high	114895	58311	114895	48.27602145	291116
Trypsin-Lys-C	133484	68079	133484	56.08665692	290657

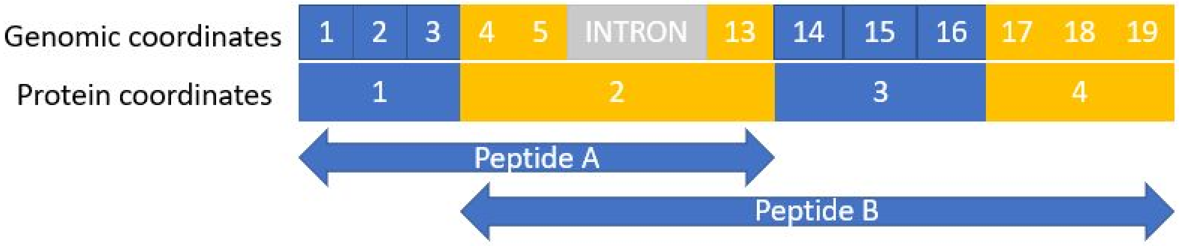

Protein coordinates	Genomic coordinates
1-2	1-13
2-4	4-19

Figure S1. Illustration example of relative peptide coordinates and their corresponding coordinates on the genome. The top row of numbers represents nucleotides in the genome, with each triplet corresponding to one amino acid in the protein sequence. The row underneath represents the relative protein coordinates in amino acids (alternating blue and yellow).
